# Multiobjective optimization framework for designing a steering system considering structural features and full vehicle dynamics

**DOI:** 10.1038/s41598-023-45349-z

**Published:** 2023-11-09

**Authors:** Carlos Llopis-Albert, Francisco Rubio, Carlos Devece, Shouzhen Zeng

**Affiliations:** 1 Instituto de Ingeniería Mecánica y Biomecánica (I2MB), Universitat Politècnica de València (UPV), Camino de Vera S/N, 46022 Valencia, Spain; 2 Department of Business Organization, Universitat Politècnica de València (UPV), Camino de Vera S/N, 46022 Valencia, Spain; 3 School of Business, Ningbo University, Ningbo, 315211 China

**Keywords:** Mechanical engineering, Applied mathematics, Computational science, Software

## Abstract

Vehicle handling and stability performance and ride comfort is normally assessed through standard field test procedures, which are time consuming and expensive. However, the rapid development of digital technologies in the automotive industry have enabled to properly model and simulate the full-vehicle dynamics, thus drastically reducing design and manufacturing times and costs while enhancing the performance, safety, and longevity of vehicle systems. This paper focus on a computationally efficient multi-objective optimization framework for developing an optimal design of a vehicle steering system, which is carried out by coupling certain computer-aided design tools (CAD) and computer-aided engineering (CAE) software. The 3D CAD model of the steering system is made using SolidWorks, the Finite Element Analysis (FEA) is modelled using Ansys Workbench, while the multibody kinematic and dynamic is analysed using Adams/Car. They are embedded in a multidisciplinary optimization design framework (modeFrontier) with the aim of determining the optimal hardpoint locations of the suspension and steering systems. This is achieved by minimizing the Ackermann error and toe angle deviations, together with the volume, mass, and maximum stresses of the rack-and-pinion steering mechanism. This enhances the vehicle stability, safety, manoeuvrability, and passengers’ comfort, extends the vehicle systems reliability and fatigue life, while reducing the tire wear. The method has been successfully applied to different driving scenarios and vehicle maneuvers to find the optimal Pareto front and analyse the performance and behaviour of the steering system. Results show that the design of the steering system can be significantly improved using this approach.

## Introduction

The automotive industry is undergoing a period of change because of the digital transformation, the circular economy, and the sustainable development goals^1–4^. Moreover, manufacturers are struggling to meet these challenges in the context of a highly competitive and rapidly changing marketplace. In this sense, the smart manufacturing in the automotive industry by means of CAE software has matured rapidly in recent years with the aim to assess the full-vehicle dynamic behaviour during the design, development, and prototype stages^5^. Compared to traditional experimental procedures, this allows to reduce costs and design and production times while maximizing vehicle performance, comfort, environmentally friendly features, quality, and safety^6^.

The suspension and steering systems play a major role to achieve a good compromise between the vehicle handling, stability, safety, and ride comfort^7^. This is because they are conflicting goals, in which the achievement of one objective is hindered by others. Therefore, there is a trade-off between vehicle handling and ride comfort. In this sense, a superior handling quality implies that the vehicle attains the state of motion required by the driver more quickly and accurately, while a superior stability means that the vehicle is capable of rapidly restoring the original state of motion under external forces and moments. Therefore, an appropriate understanding and design of the crucial parameters associated with the two systems is indispensable.

Several efforts have been made to come up with the best solutions. In this sense, the literature has addressed the design of the suspension and steering systems using several methods (e.g.^8–15^). They encompass approaches in which well-known vehicle system dynamics simulation packages or analytical equations are used to simulate, optimize, or perform sensitivity analysis for the estimation of parameters that affect vehicle dynamics behaviour. Moreover, some authors have also coupled either analytical equations that model the dynamic behaviour of vehicle systems or CAE tools with optimization methods, such as evolutionary or genetic algorithms for the same purpose (e.g.^16–19^).

For instance, the Adams/Car (Automated Dynamic Analysis of Mechanical Systems) software was used to simulate the kinematics and compliance testing of a virtual suspension system prior to conducting prototype experiments^20–23^. A sensitivity analysis using this software was carried out to optimize vehicle parameters by changing the suspension and steering hardpoint locations on the full-vehicle dynamic characteristics of a passenger car to implement vehicle power, such as AFS (Adaptive Front-lighting System), ECS (Electronic Control Suspension system), 4WD (4-Wheel Drive)^24^. Other authors have also optimized the front suspension and steering parameters of an off-road vehicle using Adams/Car simulation^21,23^. An analysis of the kinematics and compliance of a passive suspension system using Adams/Car was presented by^22^. The vehicle cornering performance evaluation and enhancement based on CAE and experimental analyses was investigated by^5^. A multi-objective optimization framework for designing a vehicle suspension system together with a comparison of the results using different algorithms was carried out in^6^.

Additionally, several analytical approaches based on Ackermann theory were used to analyse and control critical suspension properties^25^, while several equations were proposed for relating parameter measurements with the optimized Ackermann steering geometry^26^. Attempts to relate the inside and outside steered wheels of a vehicle were made to correct the theoretical equation for the Ackermann steering angle based on experimental results^27^. The effects of the steering geometry and compliance, together with the steering linkage friction and tire static friction torque on the steering effort was also examined^28^. Moreover, a methodology was proposed for fine tuning the design and dimensions of the steering linkages with the aim to minimize the steering effort while satisfying the imposed restrictions on the hardpoint locations. The repercussion of dynamic load changes on the vehicle steering geometry and the resulting non-uniform tire wear of the front steered wheels was also investigated^29^. The optimal kinematic design of a multi-link steering system for a bus independent suspension was achieved by means of the application of Response Surface Methodology (RSM)^30^. The optimum suspension hardpoint locations for attaining steering returnability in vehicle, while an improved design of steering system by reducing the steering ratio were introduced, respectively, by^31,32^.

To sum up the literature has proven that the employment of CAD/CAE software leads to key benefits in the design, development, and prototype stages of a vehicle. This paper goes a step further in the current literature by coupling powerful CAD/CAE tools that are integrated within an optimization framework for an efficient design of vehicle systems. It poses a multi-objective optimization problem that considers the geometrical features and material properties of vehicle systems, the full-vehicle kinematics and dynamics behaviour, and an analysis of the structural integrity of the key components of the steering system. It takes into account a broad-spectrum of design variables, objectives, and restraints that has been successfully applied to different driving scenarios and vehicle maneuvers. Furthermore, the framework compares different optimization strategies, which comprise heuristics optimizers, evolutionary algorithms, multistrategy optimizers, and gradient-based algorithms. The rest of the paper is organized as follows. First, a brief description of the steering and suspension systems and their key parameters influencing the vehicle dynamics are presented. Subsequently, the methodology and the optimization framework are described. Then, the methodology is successfully applied to different driving manoeuvres, in which the optimization framework finds a suitable compromise between the design variables. Finally, the findings and recommendations are discussed.

## Materials and methods

### Suspension and steering systems

An in-depth review of the theoretical foundations with regard to vehicle suspension systems can be found in^33^, on the steering mechanism in^34,35^, while on the vehicle dynamics problems and its corresponding handling, stability and ride comfort in (e.g.^16,17,19,36^). These systems play a major role in the vehicle handling and ride comfort. In fact, the main factors affecting the ride comfort are the stiffness of the suspension components (e.g., shock absorbers, springs, anti-roll bars and bushings), the suspension geometry, vehicle mass and weight distribution. Ride comfort prevents passengers from certain harmful effects on body because of vehicle vibrations and is subject to specific regulations. They cover conditions such as back pain, osteoarthritis, hyperventilation, disc slippage, etc.^37^. The regulations states that ride comfort is dependent on the root mean square (RMS) values of the sprung mass acceleration and the frequency of the body's vibrating. These frequencies can be divided into two categories: those that are unpleasant or dizzying (0.5–80 Hz) and those that are healthy and relaxing. (0.1–0.5 Hz). Road holding, on the other hand, refers to a vehicle tire's capacity to maintain contact with the road surface.

They also influence the vehicle handling (i.e., how a wheeled vehicle reacts and responds to a driver's inputs), which refers to how a vehicle performs specially during cornering, acceleration, and braking as well as on the vehicle's directional stability when moving in steady state condition. The vehicle stability is commonly supported by computerized technology, which allows detecting and reducing loss of traction (i.e., skidding). The purpose of these vehicle systems, their elements and key design parameters that influence vehicle dynamics are subsequently explained.

The suspension system connects the wheels to the vehicle chassis and is made up of several mechanical linkages, springs, and dampers. It transmits the forces and moments between the sprung and unsprung mass, which allow absorbing and damping the impacts and vibrations induced by surface irregularities and dynamic loads while preserving passengers’ ride comfort and vehicle handling and stability. It is also responsible for keeping the wheels in contact with the ground, while minimizing the loss of traction, lateral movement because of centrifugal forces, and tire wear. A high-performance suspension system must consider a wide range of factors. It should allow a movement of a wheel to be independent from the other wheel of the axle and provide structural efficiency by properly transferring the mechanical loads from the suspension system to the vehicle chassis. It should lead to a satisfactory isolation of road irregularities to improve passengers’ comfort, for which a fine tuning of the stiffness and damping coefficients of springs and shock absorbers is required. The suspension system is intended to mitigate the anti-dive phenomenon in which the front of the vehicle dips and the tail rises and the anti-squat (opposite response) in order to increase passengers’ comfort. They occur in the breaking and acceleration events, respectively. The geometry of the suspension system affects the vehicle body roll motion and its lateral behaviour during cornering manoeuvres so that it should be optimized.

The steering system allows a directional control of the vehicle in accordance with the driver’s preference by controlling and directing the steering wheel, which rotation is converted into a linear motion by the rack and pinion and eventually transmitted into the wheels by the tie roads and steering arms connected to the uprights by ball joints that can bear harsh road conditions. Additionally, both vehicle systems should be designed with low weight by using optimal geometry and lightweight materials to reduce the transmitted shocks to the chassis, long fatigue life and low cost.

**Figure 1:**
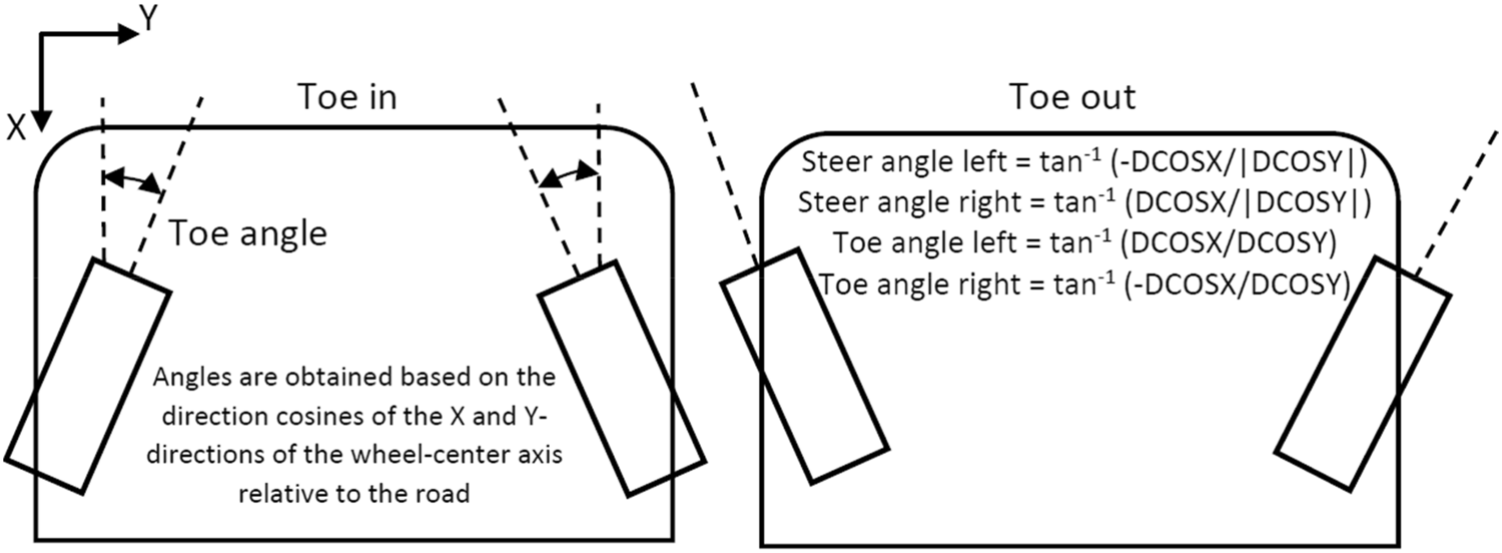
Representation and calculation of the toe angle, which is a design variable optimized in the multi-objective optimization process.

**Figure 2:**
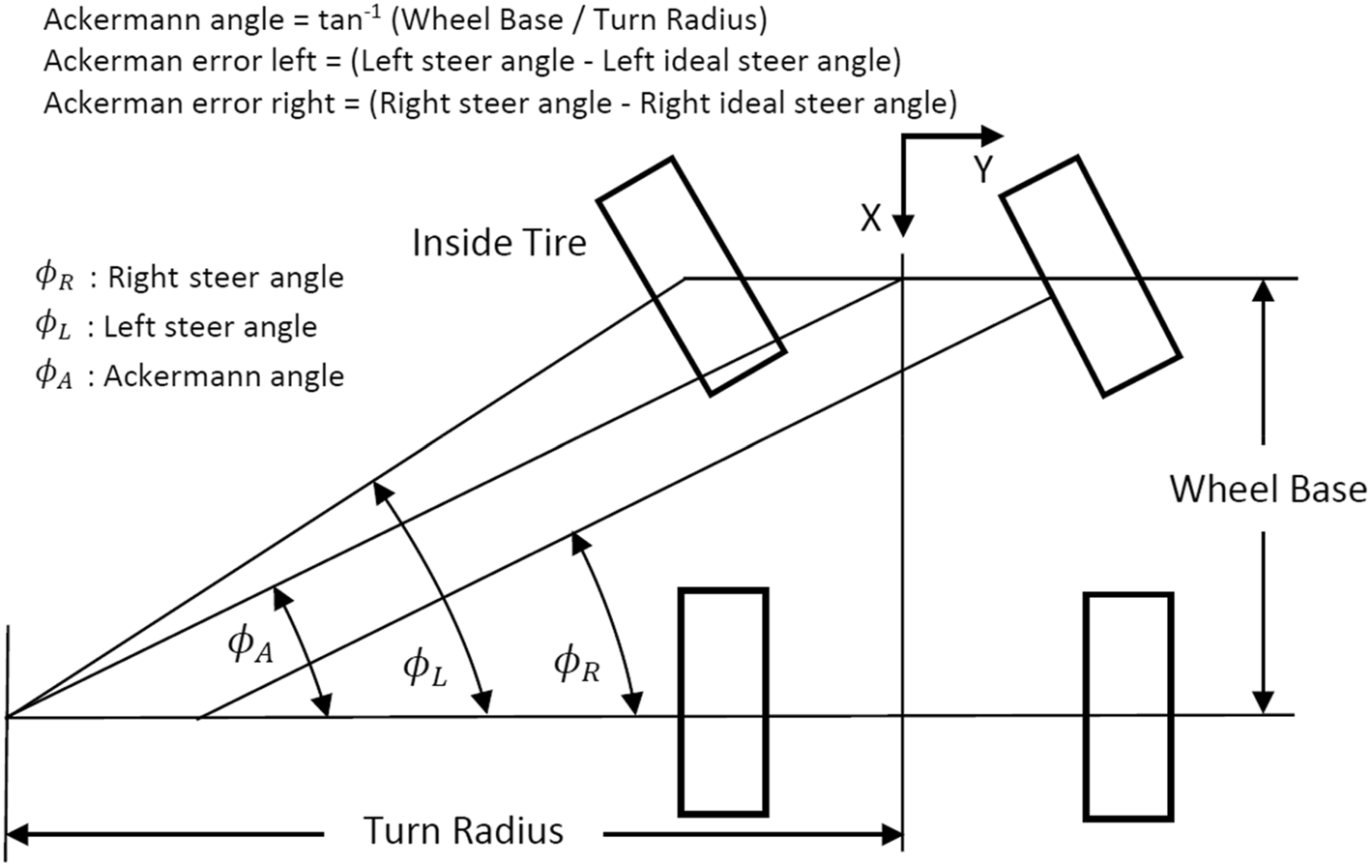
Representation and calculation of the Ackermann error, which is a design variable optimized in the multi-objective optimization process.

Suspension and steering systems depend on a wide set of parameters that affect the orientation of the wheel regarding the ground and the performance of the vehicle while braking, acceleration and cornering. These wheel alignment settings cover among others the camber, caster and toe angles, scrub radius, track width, and kingpin inclination angle. They can be modified by changing the hardpoint locations and angles of the fixations of the mechanical linkages on the chassis and the uprights. The suspension system, which rotates to permit wheel motion, is what connects the wheel to the chassis. Therefore, maintaining ideal parameter values within the preferred limits becomes more difficult the longer the travel of the wheels because of the road irregularities and forces applied. Consequently, it is of vital importance to carry out an optimization of these parameters to keep them within the limits established and improving vehicle handling and driving stability. The design variables optimized in the multi-objective optimization procedure and their most correlated parameters that affect vehicle dynamics are subsequently presented (Figs. 1 and 2):

- Toe angle is the angle between the longitudinal axis of the vehicle and the line of intersection of the wheel plane and the vehicle's XY plane. It is positive if the wheel front is rotated in towards the vehicle body. It has a great impact on the vehicle performance, e.g., on straight line stability, steering and acceleration. It is of vital importance to minimize toe angle variation during wheel travel to avoid bump steer. This occurs when the wheel steers itself without input from the steering wheel. By optimizing the hardpoint locations the bump steer can be minimized. Additionally, toe angle is used to compensate for understeer or oversteer. Common values of the toe angles are between − 1/4 inch plus or minus 1/8 inch.
- Ackermann angle is the angle whose tangent is the wheelbase divided by the turn radius. Ackermann angle is positive for right turns. Ackermann steering refers to the geometric configuration mainly designed as a trapezoidal linkage, due to its simplicity and ease of manufacturing, whose purpose is to avoid tyre sliding by compensating the effect that the outer and inner wheels travel circumferences of different radii when cornering.
- Ackermann error is the difference between the steer angle and the ideal steer angle, which gives an Ackermann steer geometry or 100% Ackermann. Since ADAM/Car uses the inside wheel to calculate the turn centre, the Ackermann error for the inside wheel is zero. A positive Ackerman error means that the actual steer angle is greater than the ideal steer angle or the actual is steered more to the right. Ideal steer angle (i.e., 100% or pure Ackermann) means the linkage extensions are cut in the centre of the rear axle. If the percentage is greater than 100, they will be cut in front of the rear axle; and behind if it is lower. Then percent Ackermann is the ratio between the actual and the ideal Ackermann expressed as a percentage. In other words, in an Ackermann steer geometry the wheel-centre axes for all four wheels pass through the turn centre. A pure Ackermann steering system is not used in modern vehicles. On the contrary, anti-Ackermann steering is designed for high-speed cornering, where cornering forces are high, in order to improve the steering response by reducing slip and providing more grip to the outside tire during larger radius turning corners. As Ackermann increases, more forces and inertia are held in the steering linkages, which can enhance the steering system ability to self-return. In theory, this should increase returnability.
- Steering systems inner and outer tierods: they transmit force from the steering wheel to the front wheels, allowing the driver to change directions. They are attached to the steering rack and connect the steering gear to the steering knuckle. They are an essential component of the vehicle steering system that, if they become worn or loose, could lead to tire wear and handling problems.

### Methodology

The design of a vehicle system demands the use of complex optimization-simulation techniques that allows estimating the crucial parameters and their impact on the vehicle dynamic behaviour. The methodology presents an efficient design and representation of a real-world vehicle steering and suspension systems by posing a multi-objective optimization framework to come up with a good compromise between the design variables and including a wide range of advantages beyond traditional engineering methods:

- Enables an automated simulation by coupling powerful engineering tools from a single workflow and evaluates thousands of designs.
- Considers the full-vehicle kinematics and dynamics behaviour, together with the structural integrity analysis of main mechanical components.
- Deals with a wide range of explanatory and response variables.
- Takes into account the geometric characteristics and material properties of the steering and suspension systems.
- Includes different driving scenarios entailing diverse vehicle dynamics manoeuvres.
- Allows conducting testing procedures, monitoring, and maintenance of vehicle systems.
- Integrates advanced data analysis to derive the Pareto optimal front and balance conflicting objectives in order to gain deeper insight into design alternatives and make informed decisions.
- Easily extendable to the design of other vehicle components.
- Maximizes efficiency and reduces operating costs.

**Figure 3:**
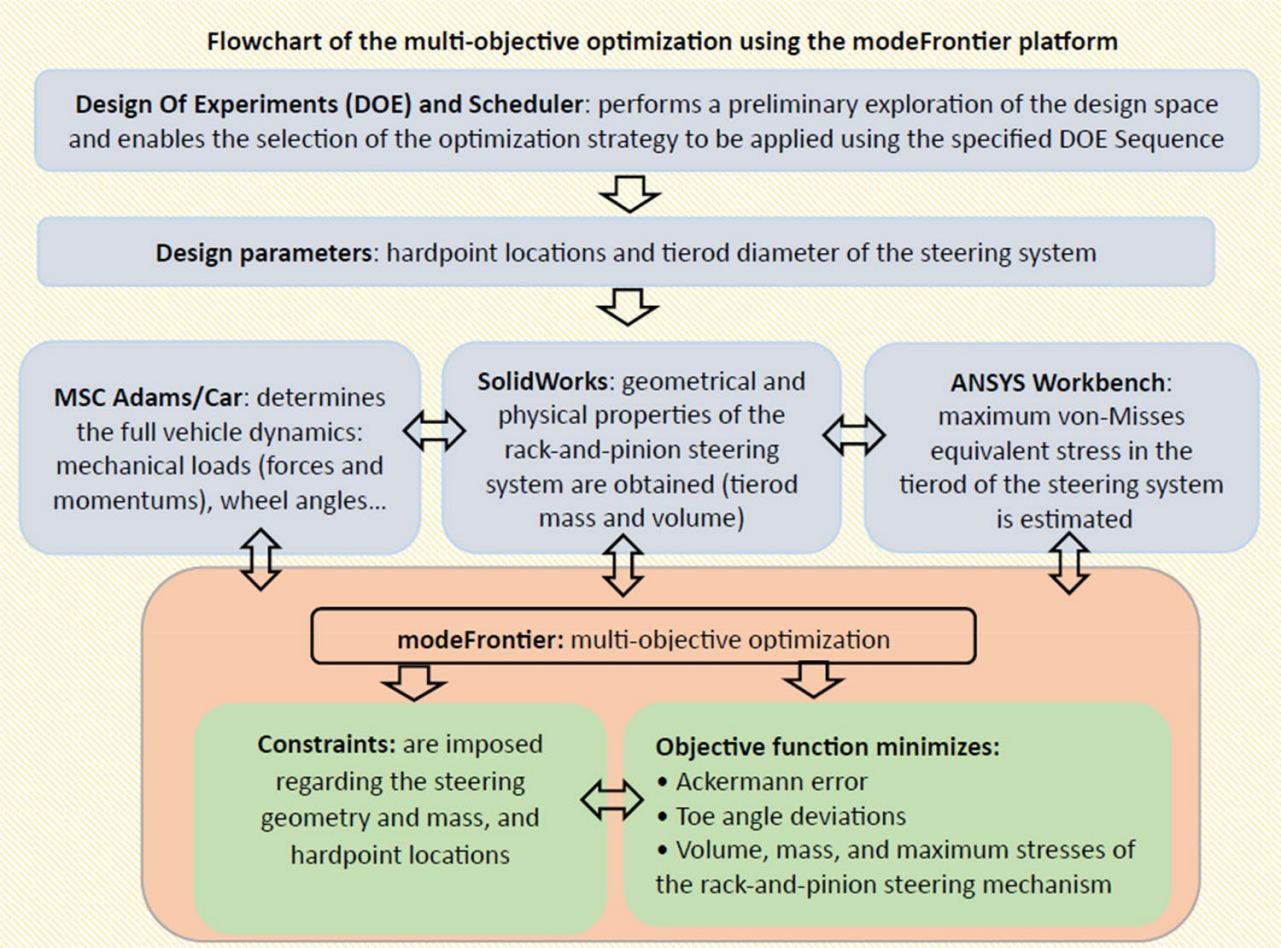
Flowchart of the optimization framework illustrating the links between the CAD/CAE tools and the flow of information of the multiphysics processes.

Figure 3 displays the flowchart diagram of the multi-objective optimization framework. It shows the flow of information between the different computer-aided design (CAD) and computer-aided engineering (CAE) software programs. This digital representation of the system allows to adequately simulate the behaviour of the mechanical components and the involved multiphysics processes. The methodology includes creating a 3D CAD model of the steering system based on a rack-and-pinion mechanism in SolidWorks, that allow to optimize the diameter of the tierod, its mass and volume. Moreover, an analysis of the structural integrity of the steering system using Ansys Workbench is performed and a multibody kinematic and dynamic analysis of the full vehicle performance is carried out by means of Adams/Car. Eventually, all results are embedded in a multidisciplinary optimization design framework (modeFrontier) with the aim of determining the optimal hardpoint locations (hpl) of the suspension and steering systems. In this sense, Adams/Car is used to determine wheel alignment settings such as the Ackermann error and toe angle deviations, together with the mechanical loads (forces and momentums) that are applied to the tierod of the steering system. Afterwards, these loads are transferred to ANSYS to estimate the maximum von-Mises equivalent stresses (SEQ) that fulfils the constraints based on the geometry, material properties and loads applied. This study considers a homogeneous material for the steering system based on structural steel with tensile yield strength of 250 MPa, compressive strength of 250 MPa, a tensile ultimate strength of 4.6E + 08 Pa, and a density of 7850 kg/m^3^. In this way the methodology allows the steering system to support the existing loads, while reducing vibrations and increasing its fatigue life.

Then the multi-objective optimization procedure minimizes the Ackermann error and toe angle deviations, together with the volume, mass, and maximum stresses achieved in the rack-and-pinion steering mechanism based on the full vehicle dynamics. The methodology relies on an initial set of parameters that are updated during the optimization procedure, which cover the hardpoint locations as well as the stiffness of the spring, bushing, and damping characteristics of the suspension system. Hardpoints are locations that connects the steering and suspension systems to the vehicle body. They play an essential role in the vehicle dynamics characteristics, since they determine certain parameters such as the described wheel angles, the longitudinal and lateral forces and moments on the wheel and load transfer. Therefore, the optimization of the hardpoint locations is justified with the aim of enhancing the vehicle handling stability and ride comfort, finding a suitable compromise between them, and analysing the corresponding trade-offs. The optimal set of parameters also allows to design mechanical components with higher vehicle systems reliability and fatigue life while reducing the tire wear.

Note that apart from the optimized parameters, which cover the Ackermann error and toe angle deviations, there are other key parameters that influence the steering and wheel alignment. The selection of these parameters is because they play a major role in the dynamics of the vehicle. The other parameters comprise the camber angle, caster angle, Kingpin inclination, steering angle, slip angle and the wheel instantaneous roll centre. For the sake of conciseness, only the parameters that are optimized have been explained in the previous section, while a deep understanding of the other parameters and their effect on the vehicle dynamics can be found, for instance, in the documentation associated with the Adams/Car. Furthermore, the use of more parameters in the objective function would lead to an overparameterized optimization problem.

The multi-objective optimization framework in modeFrontier allows to perform the optimization using either local (commonly gradient based), global (commonly non-gradient based or evolutionary) algorithms or a combination of or a combination of both depending on each problem. Furthermore, different approaches can also be applied, which cover heuristics optimizers, multistrategy optimizers, evolutionary algorithms, and gradient-based algorithms. An in-depth explanation of such approaches can be found in^29^. In addition, since the methodology entails the design of 3D mechanical components, full vehicle kinematics and dynamics and the calculation of stresses and deformations of mechanical components using the finite element method (FEM), the reader is referred to the specialized literature and software user documentation for a detailed explanation of the underlying multiphysics processes, theory and modeling aspects of the embedded engineering tools. For the sake of conciseness, note that detailed explanation of such equations is unapproachable and beyond the scope of this scientific article. It is worth mentioning that although the modeFrontier platform offers many advantages for the implementation of a multi-objective optimization problem, the reader should be aware that advanced modelling knowledge of both the platform and the embedded engineering tools and a certain degree of expertise on the underlaying physical processes are required to successfully implement an optimization problem using this platform.

## Application of the methodology to different driving scenarios

The methodology has been successfully applied to different case studies that cover full-vehicle driving scenarios, that include a wide range of manoeuvres for a complete analysis of the dynamic behaviour of the vehicle. Moreover, this allows the optimization procedure to find a suitable compromise between the design variables for these possible manoeuvres of the vehicle. In this sense, it provides a reduction in the vehicle systems development in terms of time and costs, while enhancing ride comfort and vehicle performance. The case studies are based on the multibody modelling and simulation environment Adams/Car software package. The first full-vehicle virtual test analysis covers a driving scenario that entails a cornering-event, i.e., a braking in turn event (S1); the second driving scenario involve a constant radius cornering (S2); the third deals with a straight-line braking event (S3); and the fourth tackles a roll stability event, specifically, a ramp (corkscrew) (S4). The parameters adopted in such events are displayed in Table 1. To avoid overparameterization, which could cause convergence problems and high correlations of the estimated parameters, the study focuses on the hardpoint locations (coordinates) of the outer left lower control arm of the suspension system and the corresponding tierod of the steering system. This is because these parameters present the greatest influence on the behaviour of the vehicle dynamics^5^.

**Table 1:** Driving scenarios parameters used in the simulations for a braking in turn event (S1), a constant radius cornering (S2), a straight-line braking event (S3); and a ramp (corkscrew) (S4).

Parameters for S1	Values	Parameters for S2	Values
Gear position	4	Gear position	1
Step size	0.1 s	Step size	0.2 s
Lateral acceleration	0.45 g	Maximum steer angle	180
Turn radius	30 m	Turn radius	61 m
Turn direction	Left	Turn direction	Left
Sterring input	Lock steering while braking	Shift gears	Yes
Brake deceleration	0.2 g	Duration	10 s
Maximum brake duration	2.5 s	Final velocity	80 km/h
Initial velocity	41.42 km/h	Initial velocity	10 km/h

Parameters for S3	Values	Parameters for S4	Values
End time	5 s	End time	5 s
Number of steps	50	Number of steps	50
Initial velocity	120 km/h	Initial velocity	80 km/h
Steering input	straight	Gear position	4
Start maneuver	0 s	Step size	0.1
Final brake	3 s	Road data file	2d flat
Step duration	0.2 s		
Gear position	6		
Road data file	2d flat		

On the one hand, a rack-and-pinion steering system, which convert rotation motion into linear motion, is selected because it provides a simple, compact, and robust control over the vehicle with lower cost with high mechanical efficiency. The most adverse disadvantage is due to the inherent friction, which leads to a constant wear of the mechanical components. On the other hand, a non-parallel double wishbone independent suspension system based on a four-bar guiding mechanism is chosen because its simplicity, ground clearance, impact protection, versatility in providing a wide range of motion, accuracy, and durability. Moreover, it also provides the best cornering performance, an increased negative camber gain while rolling, a vertically independently movement of each wheel on the same axle and an optimum compromise between vehicle handling and ride comfort. On the negative side, it is sensitivity to misalignment, which can affect their accuracy and performance and more expensive to design and manufacture than some other types of linkages.

A linear relationship in the front suspension system between the spring force and the displacement has been considered, which varies between ± 12,000 N and ± 100 mm, respectively. With regard to the damper force and the velocity a polynomial relationship has been defined within the interval of [4000, − 1250] N and ± 1250 mm/s, respectively.

A set of restraints are applied to the multi-objective optimization problem, which entail variation intervals for the tierod diameter, its mass, and hardpoint locations (hpl). In fact, the initial guess of the parameter set, which are presented in Table 2, can be changed during the optimization procedure within a range of 7% to maintain a good vehicle handling and stability and not to interfere with the frame and other vehicle systems. For all scenarios, an ideal Ackermann direction and minimum deviations of the toe angle with respect to − 1° are sought.

**Table 2:** Initial and optimized front hardpoints locations (hpl) of the outer left lower control arm (lca) and the corresponding tierod for the different driving scenarios. The initial guess of the parameter set is allowed to change within a 7%, which has been implemented as restrictions in the optimization problem.

Parameter (coordinates)	Initial guess (mm)	Optimized hlp for S1 (mm)	Optimized hpl for S2 (mm)	Optimized hpl for S3 (mm)	Optimized hpl for S4 (mm)
X lca_outer	267	253.65	280.35	280.35	280.35
Y lca_outer	− 750	− 787.5	− 787.5	− 787.5	− 750
Z lca_outer	130	126.75	136.5	123.5	130
X tierod_outer	417	437.85	437.85	396.15	396.15
Y tierod_outer	− 750	− 786.328	− 787.5	− 712.5	− 712.5
Z tierod_outer	330	346.5	313.5	346.5	346.5

It is important to mention that the optimization problem was carried out to withstand the maximum mechanical loads determined by Adams/Car, and that the four objectives are given equal weights in the penalty function. A Design of Experiments (DOE) has been defined using the modeFrontier platform to do a preliminary exploration of the design space, and a variety of optimization strategies are implemented by employing the given DOE sequence. A total of 500 simulations are obtained for each optimization approach and driving scenario.

**Figure 4:**
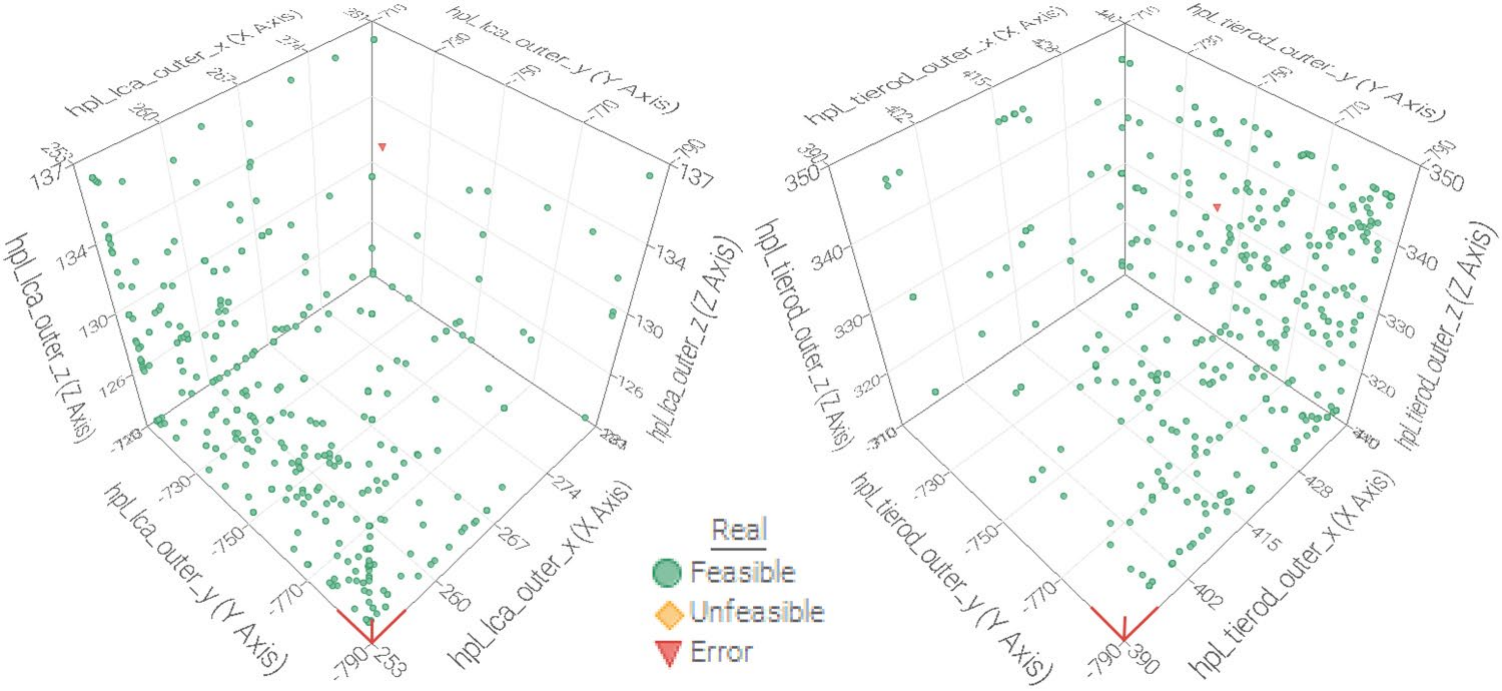
Scatter plot of the parameter values used in the optimization process regarding the outer hardpoint locations of the left lower control arm and the tierod, in which the dimensions are expressed in mm.

Figure 4 presents a scatter plot of the parameter values applied in the optimization procedure for the outer hardpoint locations of the left lower control arm and the corresponding tierod. This figure shows a wide parameter dispersion for parameters, which enables the global optimum to be reached by optimizing across the entire design space.

## Results and discussion

For each driving scenario and optimization method, five hundred simulation runs were performed. On average 98% of feasible simulations were achieved. The methodology allows obtaining a Pareto-optimal front that comprise a set of non-dominated solutions. This set optimizes the four objectives in the multi-objective optimization problem, which encompasses the Ackermann error, the toe angle deviations, together with the mass and maximum von-Mises equivalent stresses of tierod of the rack-and-pinion steering mechanism.

**Figure 5:**
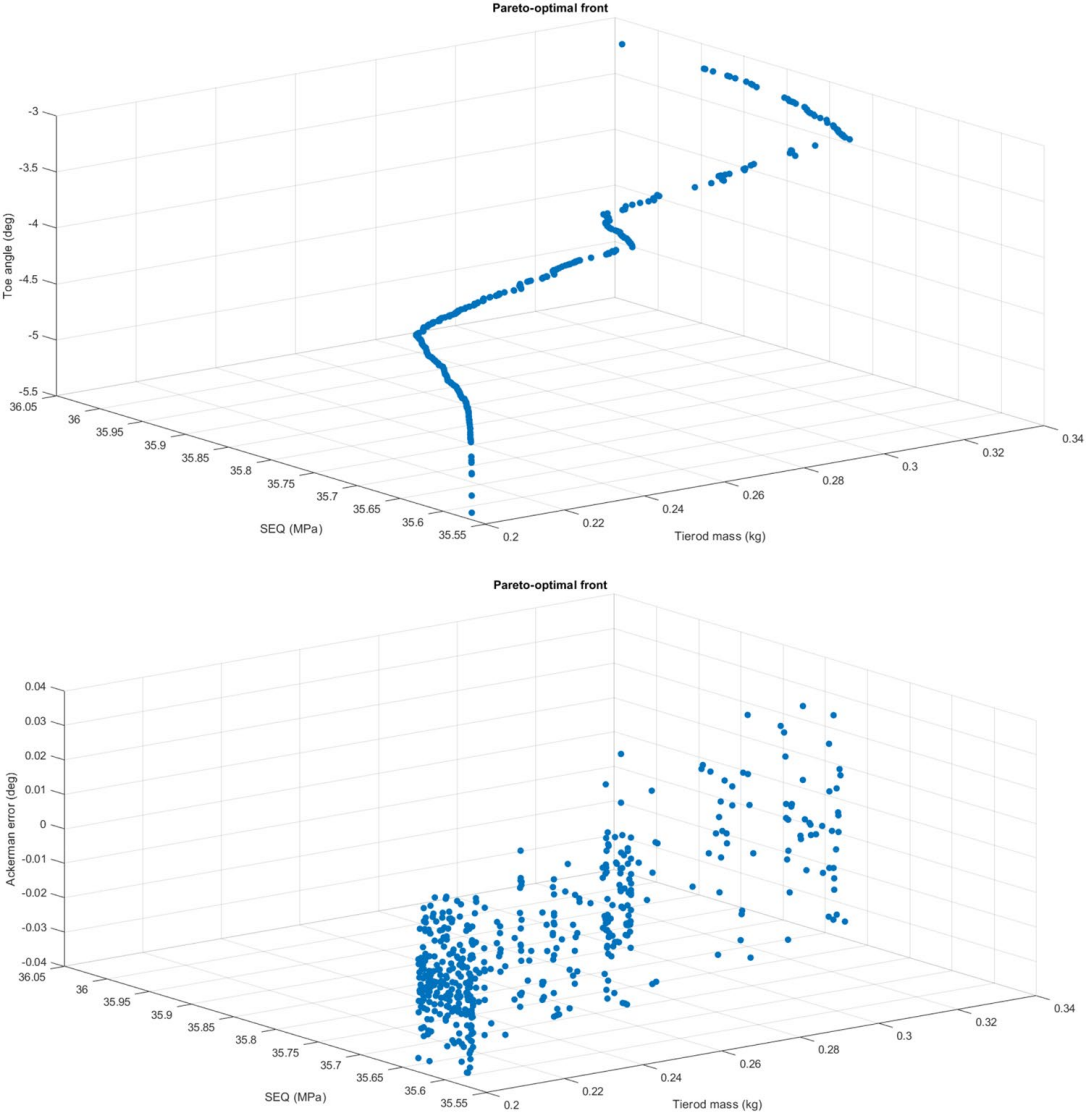
Pareto-optimal fronts set for scenario S1 presenting the feasible solutions of the four objectives, which comprise the toe angle deviations (deg), Ackermann error (deg), and mass (kg) and maximum equivalent stress (MPa) in the tierod of the steering system.

Figure 5 shows a scatter 3D plot with the Pareto-optimal front of the entire feasible search space of the objective function for scenario S1. The results are illustrated for the PilOpt algorithm because it provides better results than other algorithms. There is no better solution on all goals than those provided in the Pareto optimal front. Therefore, compared to the set offered by the Pareto optimal front, a shift in the vector of the design variables would not simultaneously improve all goals, deteriorating at least one goal. In other words, the set of non-dominated solutions exhibits some trade-offs among the conflicting goals. Then, instead of taking into the entire range of each parameter, the Pareto optimum front pays attention on a set of efficient choices and evaluates trade-offs within this set.

**Figure 6:**
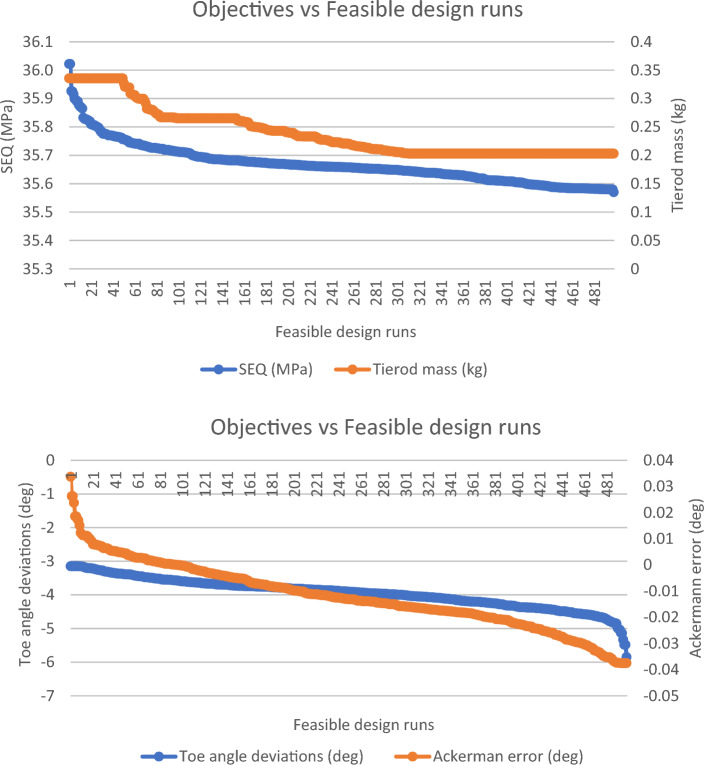
Optimal objectives values for the 500 design simulations for scenario S1 (up: maximum von-Mises equivalent stress (SEQ) and mass in the tierod of the steering system; down: toe angle deviations and Ackermann error).

A comparison between the non-optimized and optimized parameters of the vehicle systems has been carried out for the different driving scenarios (Table 2). It presents the coordinates of the hardpoint locations for the tierod and the outer left lower control arm (lca) for the different driving scenarios for both their initial guess and the optimized coordinates. Figure 6 shows for scenario S1 the reductions obtained in the objectives set after the optimization process for the different runs. A decrease of up to 39.51% for the tierod mass of the rack-and-pinion steering system, 1.25% for the maximum equivalent stress achieved in the tierod, and 85.88% for the toe angle deviations, and almost 100% reduction for the Ackermann error. Therefore, the methodology finds the best compromise solution, which leads to an optimal design of the analyzed vehicle systems. The methodology considers several competing objectives and constraints related to the geometrical and physical properties of the suspension and steering systems, and vehicle dynamics variables. Additionally, it enables a joint and multi-criteria optimization of the design variables for improving the vehicle ride comfort, handling, and stability.

The simulation findings display that the real vehicle dynamics behavior has been accurately modeled. The methodology aims to achieve a balance between the computational cost, the level of detail of the model, and the quality and precision of the results. This helps to prevent overparameterization issues caused by parameter uncertainty.

The input and output variables are related to each other. This relationship can be analyzed and understood. By examining the input and output variables, we can determine how they affect each other. Understanding this relationship is important for making predictions and improving processes.

The linear relationship between the variables is measured by means of the Pearson correlation coefficient. Because of the defined geometry and uniform density of the tierod, a perfect correlation between its volume, diameter and mass is achieved. This leads to a reduction of these design variables for the identical set of parameters. Additionally, it should be highlighted that a high Pearson correlation of 0.733 is observed between minimum values of the Ackermann error and the toe angle. A moderate correlation of the outer tierod hardpoint locations between 0.5 an 0.675 is presented for the minimum values of the Ackermann error and the toe angle. The Y coordinate of outer hardpoint location of the lower control arm exhibits a correlation of 0.485 with the toe angle deviations and 0.426 with the Ackermann error. For the rest of the variables a low correlation is observed. For instance, between the tierod mass and the Ackermann error the correlation is 0.011, while between the von-Mises equivalent stress and the tierod mass is 0.042.

**Figure 7:**
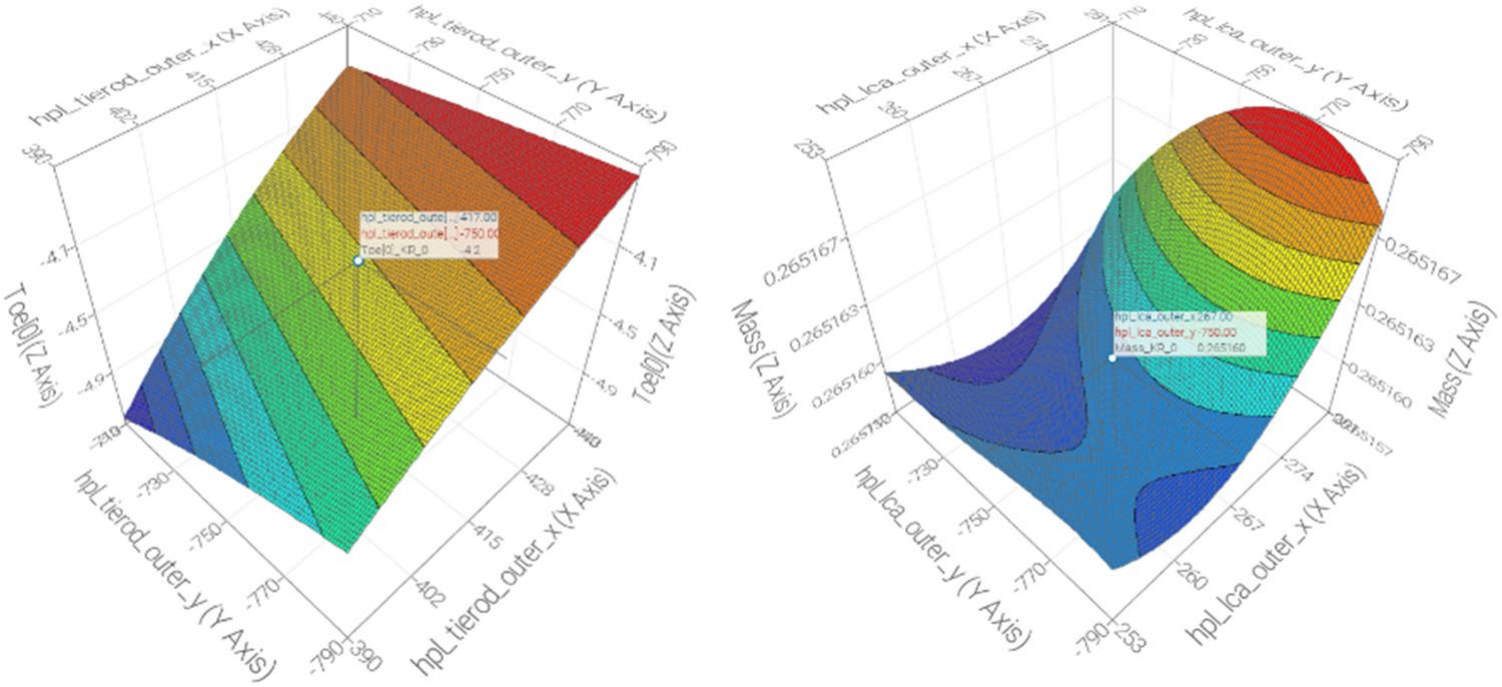
Examples of application of the RSM for analyzing the relationships between explanatory variables (hardpoints locations) and the objectives (mass an toe angle deviations in the outer tierod of the steering system).

Figure 7 displays the application of a statistical approach known as the Response Surface Methodology (RSM) for analyzing the correlations between explanatory variables (hardpoints locations) and one of the objectives (maximum equivalent stresses, Ackermann error, toe angle deviations, and tierod mass). RSM makes it possible to handle complicated real-world problems while decreasing the computational burden and the number of runs required to reach the global optimum. This is due to the fact that RSM is based on datasets rather than real-physics models (that are unaffordable in terms of processing time) in order to anticipate system reaction to an unknown configuration and perform a virtual optimization.

RSM uses Machine Learning algorithms to obtain a rapid and effective meta-model based on a dataset. This enables the validation of the model accuracy and to perform a reliable RSM-based optimization. Consequently, the use of a RSM as a surrogate model makes possible to explore the design space by means of an adequate Design Of Experiments (DOE) and to carry out the optimization procedure faster with a limited quantity of designs. In addition, a comparison of different algorithms and optimization strategies is presented.

Furthermore, it enables precise results in identifying the Pareto-optimal front, while avoiding potential downsides of each optimization technique. The optimization strategies can be divided into two groups: local (often based on gradients) and global (typically non gradient based or evolutionary). The study examines the outcomes of a wide range of optimization techniques, including gradient-based optimizers, multi-strategy algorithms, evolutionary algorithms, and heuristic optimizers^6^.

However, it is important to note the difficulties in evaluating the effectiveness of several optimization techniques^28,30^. As a result, this work performs the comparison in accordance with the advice given in the literature. The PilOpt method produced the best outcomes for the multi-objective optimization problem, as shown in Table 3. It returns the smallest value of the penalty function, rapid convergence, the largest number of feasible solutions, and a wide variety of solutions. This is due to the capability of the PilOPT algorithm to combine local and global search strategies^38,39^.

**Table 3:** Comparison of the different optimization strategies for scenario S1 regarding the minimum values achieved sof the objectives.

Objectives/algorithms	Arm mass (kg)	Maximum von-Mises equivalent stress (SEQ) (MPa)	Minimum Toe angle deviation (deg)	Minimum Ackermann error (deg)
Evolutionary algorithms				
NSGA-II	0.207	35.651	− 4.484	0.002
MOGA-II	0.203	35.726	− 3.867	− 0.013
ARMOGA	0.203	35.735	− 4.103	− 0.007
Evolution strategies	0.203	35.727	− 4.428	0.002
Heuristics optimizers				
MOSA	0.207	35.769	− 4.812	0.012
MOPSO	0.203	35.679	− 3.777	− 0.016
Multistrategy algorithms				
HYBRID	0.203	35.591	− 3.753	− 0.019
pilOPT	0.203	35.560	− 3.581	− 0.021
FAST	0.203	35.566	− 3.586	− 0.021
MEGO	0.203	35.694	− 3.366	− 0.029
Gradient-based optimizers				
NBI-AFSQP	0.203	35.759	− 4.518	0.004

It modifies the ratio between real and virtual RSM-based design evaluations to boost the performance in the process to attain the Pareto-optimal front. Since it only depends on one parameter, complex parametrization is also avoided. The values presented in Table 3 belong to the Pareto-optimal front, that illustrates a trade-off between goals. Additionally, certain methods, like the MEGO strategy, produce outcomes that are comparable to those produced by the pilOPT algorithm. Eventually, it is important to note that it will take roughly 12 h to compute 500 simulations using an 8 GB of RAM and Core i5 CPU with a 2.90 GHz clock speed.

Table 4 shows the pilOPT algorithm outcomes for the different driving scenarios, which are of the same order of magnitude and are somehow comparable. Nevertheless, different optimized values for the hardpoint locations (Table 2) and objectives (Table 4) are obtained because the vehicle is subjected to disparate dynamic settings in each of them. In this sense, since the optimal maximum stress obtained for S1 is less than for the other scenarios, the dynamics loads that appear in the tierod of the steering system for this vehicle maneuver are less demanding. As a rule of thumb, it is necessary to seek a satisfactory compromise between the design variables for all potential vehicle maneuvers and dynamic behaviors.

**Table 4:** Optimal solution attained using the pilOPT strategy for the different driving scenarios.

Driving scenario/objectives	Arm mass (kg)	Maximum von-Mises equivalent stress (SEQ) (MPa)	Minimum Toe angle deviation (deg)	Minimum Ackermann error (deg)
S1	0.203	35.560	− 3.581	− 0.0210
S2	0.203	36.690	− 2.474	0.000867
S3	0.203	36.723	− 0.659	0.00912
S4	0.203	36.916	− 0.139	0.00144

## Conclusions

A framework for optimizing the design of the vehicle suspension and steering systems has been developed, which enables an automated simulation by coupling powerful engineering tools from a single workflow and evaluates thousands of designs. The multiobjective optimization framework integrates well-proven simulation tools and considers a broad-spectrum of design variables, objectives, and restraints, which encompasses the geometrical features and material properties of vehicle systems, the full-vehicle kinematics and dynamics behavior, and an analysis of the structural integrity of the key components of the steering system. The methodology allows conducting testing procedures, monitoring, and maintenance of vehicle systems. Furthermore, it integrates advanced data analysis to derive the Pareto optimal front and balance conflicting objectives in order to gain deeper insight into design alternatives and make informed decisions.

The methodology has been successfully applied to different driving scenarios entailing a wide range of vehicle maneuvers. These case studies examine the performance of the full-vehicle dynamics by optimizing key parameters of the suspension and steering systems. Moreover, a comprehensive set of optimization strategies has been compared to accurately find the Pareto-optimal front of the multi-objective problem. Results have shown that the efficient optimization approach reduces vehicle systems design time and manufacturing costs while enhancing the vehicle performance (i.e., vehicle handling, stability, safety, and ride comfort). In addition, the fatigue life of the mechanical components is expanded. All optimization approaches obtained a great convergence, diversity of solutions, and percentage of feasible solutions, allowing for finding the Pareto-optimal front. This set of non-dominated solutions efficiently assesses vehicle systems performance and analyzes trade-offs between design parameters. It has been proved that different combinations of these factors can produce quite varied outcomes in terms of a vehicle handling, stability, and ride comfort. This demonstrates the worth of the methodology for significantly enhancing vehicle systems design, especially when compared with conventional simulation engineering approaches for vehicle development.

The proposed optimization methodology can be used as a decision support system for designers in determining the set of parameters of the suspension and steering systems to provide the requested vehicle dynamic behavior. This is because different set of parameters lead to opposing vehicle dynamics effects, so it is needed to find a suitable comprise between such parameters. Moreover, the methodology offers flexibility in the selection of trade-offs between design alternatives. As a result, it maximizes efficiency and reduces operating costs.

As part of future research, it might be possible to carry out a sensitivity analysis of the impact of additional parameters on the dynamics of the vehicle, and additional vehicle maneuvers and driving tests since they require the assessment of diverse dynamic characteristics. Additionally, the methodology can be applied to other vehicle systems such as transmission or braking systems. Moreover, it can be extended to designing any mechanical system. The only challenge lies in correctly modeling with CAD/CAE tools its geometric and material properties, the kinematics and dynamics of the mechanism, as well as the boundary conditions of each specific problem.

## Data Availability

The datasets used and/or analysed during the current study available from the corresponding author on reasonable request.
